# The genomics of t’ef and finger millet domestication and spread

**DOI:** 10.1098/rstb.2024.0196

**Published:** 2025-05-15

**Authors:** Degsew Z. Mekonnen, Ana Isabel Gomes, Rui S. R. Machado, Hugo Rafael Oliveira

**Affiliations:** ^1^ ICArEHB – The Interdisciplinary Centre for Archaeology and Evolution of Human Behaviour, University of Algarve, Faro, Portugal; ^2^ EHA - Ethiopian Heritage Authority, Addis Ababa, Ethiopia; ^3^ Centre for Marine and Environmental Research (CIMA)—Infrastructure Network in Aquatic Research (ARNET), Faculty of Science and Technology, University of Algarve, Faro, Portugal; ^4^ Faculdade de Ciências Humanas e Sociais, University of Algarve, Faro, Portugal

**Keywords:** archaeobotany, origins of agriculture, diversity, DArTSeq, Ethiopia

## Abstract

The Northern Highlands of Ethiopia and Eritrea (NHE) were a centre for food production in Africa, hosting one of the earliest agriculture-based complex societies on the continent. The NHE’s geographical connections with the Arabian Peninsula, and Nilotic cultures led to the cultivation of southwest Asian crops and African native domesticates in its territory. Additionally, the NHE were also the domestication centre for crops like t'ef (*Eragrostis tef* (Zucc.) Trotter) and finger millet (*Eleusine coracana* L. Gaertn L.), after well-adapted local wild plants. Considering the paucity of the archaeobotanical record in the region and food remains' preservation issues, in this study, we aim to investigate the domestication and spread of t'ef and finger millet using genomics and interpreting the results in the light of archaeological proxies. Our data confirmed *Eragrostis pilosa* and *Eleusine coracana* subsp. *africana* as the sole wild progenitors of t’ef and finger millet, respectively. T’ef was initially domesticated in the NHE before spreading into southern Ethiopia and eastwards into southern Arabia. Finger millet spread followed two routes: one leading eastwards through the Red Sea to India, and the other southwards, through Kenya and Uganda, reaching southern Africa.

This article is part of the theme issue ‘Unravelling domestication: multi-disciplinary perspectives on human and non-human relationships in the past, present and future’.

## 1. Introduction

The Holocene period marked the onset of the agricultural revolution, a pivotal moment in human history. This transition from hunter-gatherer lifestyles to agriculture and animal domestication has profoundly shaped human societies ever since [1–5]. The Northern Highlands of Ethiopia and Eritrea (NHE) were recognized by Vavilov as a centre of diversity and a place for independent plant domestication [6,7]. Harlan [8] later challenged the concept of diversity centres equating to domestication centres, although both recognized the region’s outstanding crop genetic diversity and the likelihood of it being the cradle of some African crops [6–9]. Various models have been proposed to explain the origins of food production and complex societies in the NHE, including migration/diffusion models from present-day Egypt or Sudan [10–14], from the south of the Arabian Peninsula [15,16] as mentioned in [17]. Evidence from rock art sites in northern Ethiopia suggests pastoralism reached the highlands around 4000 BP [18,19]. Faunal remains, including cattle, sheep and goats, dating back approximately 3500−4000 BP, have been discovered at various archaeological sites across Ethiopia, such as Lake Besaka, Danei Kawlos, Gobedra, Laga Oda, Kurub-07, Yabello and Mezber [20–25]. The NHE, particularly the Tigrai region, show the earliest evidence of agriculture associated with the early phases of the Pre-Aksumite culture [26–29]. Crop plant remains highlight the existence of early agro-pastoralist communities with an agricultural system integrating southwest Asia and other African crops with indigenous domesticates [29–33].

Plant species believed to have been domesticated locally include cereals such as t’ef (*Eragrostis tef* (Zucc.) Trotter) and finger millet (*Eleusine coracana*), enset (*Ensete ventricosum*), khat (*Catha edulis*), coffee (*Coffea arabica*), okra (*Abelmoschus esculentus*), noog (*Guizotia abyssinica*), gesho (*Rhamnus prinoides*) and kosso (*Hagenia abyssinica*) [8,9]. T’ef and finger millet are the principal of these crops, still being widely cultivated in Africa and parts of India where they are a component of sustainable farming systems as well as gastronomical traditions. Little is known about their domestication process, other than their putative wild progenitors. It is still not known how many times they were independently domesticated, where exactly, if their domestication process was quick or protracted and the routes by which it would then spread. T’ef is an allotetraploid (2*n* = 4× = 40) within the Poaceae family [34,35]. Morphological, cytological and molecular investigations point to *Eragrostis pilosa* (L.) P. Beauv. as the wild progenitor [34,36–40]. The morphology and taxonomical status of *Er. tef* within the genus have been widely described [41]. Classified in the same sub-family as t’ef (Chloridoideae) of grasses, finger millet (*El. coracana* L. Gaertn L.) is also an allotetraploid (2*n* = 4× = 36, AABB), cultivated widely in Africa and southern Asia, Ganapathy [42] has reviewed its taxonomical status and morphology. *Eleusine coracana* subsp. *africana* is considered its closest wild relative as they are completely cross-compatible and produce fertile hybrids [43–45]. The biology and systematics of this species and the *Eleusine* genus have been reviewed by Neves [46]. *Eleusine coracana* subsp. *africana* can be found in different parts of Africa as well as the Arabian Peninsula. Gene flow also occurs with other closely related diploid species, such as *Eleusine indica* and *Eleusine floccifolia*, these possibly being the AA and BB genome donors of the tetraploid forms [47]. The subsp. *africana* has two wild races, *africana* and *spontanea*, while subsp. *coracana* has four cultivated races; *elongata*, *plana*, *compacta* and *vulgaris* [48]. A morphological description of *Er. pilosa* and its differences from t’ef is provided by [49]. T’ef and finger millet microbotanical/macrobotanical remains were found at the Mezber site and dated to around 3500 BP, making them part of the earliest farming systems in Sub-Saharan Africa [29,50,51]. However, the wild or cultivated status of these remains cannot be determined with certainty.

Genetic studies on these two crops focus on developing markers useful for breeding of drought-tolerant, disease-resistant and higher yield varieties [34,35,40,45,52–56]. Although both species have had their genomes sequenced [57,58], to our knowledge, no genetic study has looked into their domestication history.

Genomic analysis of modern plants has been used to elucidate the history of cereals [59–61], legumes [62,63] and other crops [61,64]. Next-generation sequencing technologies coupled with complexity-reduction methods allow the cheap and quick identification of thousands of single-nucleotide polymorphisms (SNPs) in several individuals of the same species. These are ideal for the identification of population structure within crops and for determining genetic relationships between species within the same genus. One such technology is DArTSeq, which identifies genome-wide SNPs without the need for a reference genome [65,66]. DArTSeq shows better efficiency, cost-effectiveness and suitability for large-scale projects across diverse species within a genus. This approach encompasses a broader range of genomes and necessitates reduced time and effort for library preparation. Other double digestion methods for genomic complexity-reduction (such as ddRAD) yield satisfactory results; however, they may require additional procedural steps, exhibit enzyme dependency for genome coverage, potentially omit certain genomic regions owing to their targeted nature and are typically more expensive. This makes DArTSeq particularly suited for diversity and evolution studies in non-model organisms. Plant analysed using this method include wheat [67], maize [68], sugarcane [69], cowpea [70], cassava [71], pearl millet and pigeon pea [72].

Here, we report the genotyping of wild and landrace varieties of t’ef and finger millet using DArTSeq technology. The SNPs identified were used to map the geographical distribution of genetic diversity and thus infer patterns relevant to the domestication and spread of these crops. We considered these species together owing to their local origin and because both were part of the early agriculture in the NHE, leading us to hypothesize that they might share similar histories. This complements the archaeobotanical data for these species available for the NHE.

## 2. Material and methods

### (a) Plant material

A total of 94 accessions of *Eragrostis* and 46 accessions of *Eleusine*, including both wild and cultivated forms, were ordered from the United States department of agriculture-Germplasm Resources infromation Network (USDA-GRIN -United States) and Genebank Information System /Internet (GBIS/I -Germany). Accessions were selected based on the availability of seeds for distribution by germplasm banks, including as many wild accessions as possible, and covering the geographical range of these species. Among the t’ef accessions, 63 were cultivated landraces, while the wild accessions included *Er. pilosa* (*n* = 14), *Eragrostis cilianensis* (*n* = 3), *Er. cilianensis* subsp. *staros* (*n* = 3), *Eragrostis tenuifolia* (*n* = 6), *Eragrostis tremula* (*n* = 4) and one undetermined *Eragrostis* spp. accession. For finger millet, *n* = 39 accessions of the cultivated form *El. coracana* were used, while the wild accessions consisted of *El. coracana* subsp. *africana* (*n* = 4) and *El. floccifolia* (*n* = 3). The latter was added to investigate the possibility of it having introduced alleles into the crop’s gene pool through introgression. Seeds were sown in plastic vials with a mix of soil and perlite and placed in a greenhouse at the University of Algarve, Portugal. The complete list of accessions is used is provided in the electronic supplementary material, S1.

### (b) DNA extraction and DArTSeq genotyping

Fresh leaves from each accession were harvested four to six weeks after planting and crushed in liquid nitrogen. DNA was extracted with the DNeasy Plant Pro Kit (Qiagen), following the manufacturer’s instructions. DNA was quantified using a Qubit® dsDNA HS assay on a Qubit 2.0 fluorometer. DNA integrity was visually assessed through electrophoresis on a 1% agarose gel. A 96-well plate containing *Eragrostis* DNA (each well containing 300 ng of DNA) and half a plate (48 wells) containing *Eleusine* DNA, were shipped to the Diversity Arrays Technology Laboratories, University of Canberra (Australia) for DArTSeq analysis. DNA was digested using two restriction enzymes, MseI and PstI and libraries prepared for sequencing in an Illumina Hiseq2500/Novaseq6000 platform.

### (c) Data analysis

Following the *Eragrostis* DArTseq (1.0) panel, t’ef reads were mapped to the *Eragrostis* reference genome (Eragrostis tef V3—CoGe Genome ID:54599). For finger millet, a contig-based approach was used with proprietary DArT analytical pipelines. Quality control parameters were based on [73] and included the reproducibility of 100%, the overall call rate of 95% (percentage of valid scores in all possible scores for a marker) and a Q-value above 2.5. DArT™ assays comprised SilcoDArT (scores for presence/absence (dominant) markers) and SNPs found in the aligned or mapped reads (DArT SNPs). SilicoDArT markers are scored in a binary fashion, representing genetically ‘dominant’ markers, with ‘1’ = presence and ‘0’ = absence of a restriction fragment with the marker sequence in the genomic representation of the sample. In SNP data format, each allele is scored in a binary fashion (‘1’ = presence and ‘0’ = absence) and heterozygotes are scored as ‘1/1’. Both types of data were analysed using the dartR package (v. 2.9.7) for the programming language R [74]. The dartR objects were converted to genlight objects for manipulation in the R package adegenet v. 2.0.1 [75] for downstream analysis, including genetic diversity calculations. After an exploratory data analysis, quality filters were applied namely: (i) removal of monomorphic loci; (ii) minor allele frequency (MAF) > 0.05; (iii) call rate > 0.95; and (iv) read depth >8×. Code used is provided in the electronic supplementary material, S2.

### (d) Population structure

To explore similarities between the accessions in the dataset and infer population structure with non-parametric methods, we used adegenet to perform principal component analysis (PCA), calculate genetic distance matrices and compute phylogenetic trees. Results were visualized with the ggplot2 (v. 3.5.1) R package [76]. Groups identified with these methods were re-analysed separately: accessions were sorted in the raw DArTSeq files and the same quality filters previously described were applied.

For parametric analyses, population structure was examined using the Bayesian model-based clustering algorithm Structure 2.3.4 [77]. Structure was run for the two sets of accessions *Eragrostis* (*n* = 94) and *Eleusine* (*n* = 46) with *K*-values between 1 and 20, 20 000 burn-in, 40 000 Markov chain Monte Carlo iterations (MCMC) and 10 independent runs for each value of *K*. The most likely values of *K* were chosen based on the Δ*K* method [78], computed in Structure Harvester [79]. Q-matrixes were plotted in Microsoft Excel and displayed on geographical maps using the ‘geopandas’ library [80] for the Python 3.12 language (electronic supplementary material, S2) and QGIS 3.28.3 software. To explore historical or environmental effects on t’ef population structure, for those accessions with passport data reporting the geographical coordinates of collection site, we obtained environmental variables including soil type, administrative region, climate zone, Köppen climate classification and local geology (electronic supplementary material, S1). PCA plots were then coloured according to these categories to see if these corresponded to accession clusters.

Genetic diversity measures for taxa and groups identified by population structure analysis were calculated in R using the packages adegenet and hierfstat [81], based on DArT SNP datasets.

## 3. Results

### (a) Eragrostis

**Figure 1.**
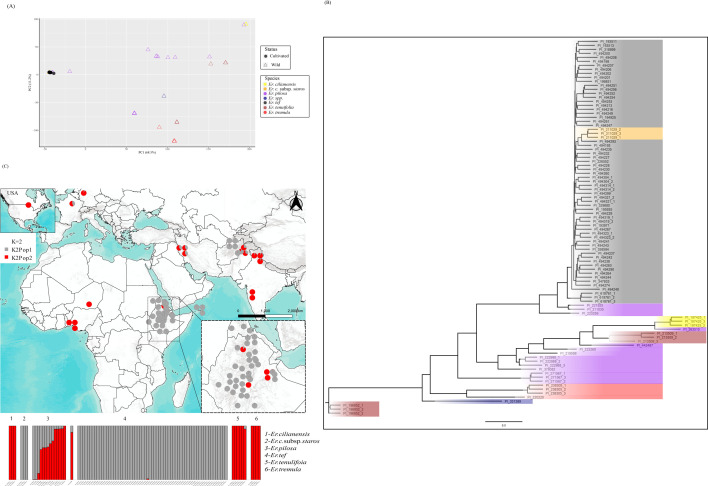
Population structure of 94 wild and domesticated *Eragrostis* accessions, based on 4455 DArTSeq SNPs. (A) First and second principal components of a PCA. Each point is an accession, the closer the points are the more genetically similar they are. Accessions are coloured according to the taxonomical classification reported in the germplasm bank’s passport data. (B) NJ phylogenetic tree based on pairwise genetic distances calculated in the R package adagenet. (C) Graphical representation of a Q-matrix of the Structure *K* = 2 model. Vertical lines correspond to accessions, and they are apportioned according to the proportional membership of each one of the two modelled clusters, or the proportion of alleles each accession gets from a hypothetical ancestral population.

The DarTSeq method uncovered 80 492 SNPs for the *Eragrostis* set, from which (after quality filtering for coverage, MAF and missing data thresholds) 4455 SNPs were retained. The DArTSilico set contained 4 32 568 markers from which 28 662 were kept after filtering. PCAs for both types of data were broadly coincident (figure 1; electronic supplementary material, figure S1). Replicate individuals within the same accessions were invariably clustered together validating the method’s ability in determining genetic relationships between plants. PCAs based on both marker types also split the accessions into three groups. One included all the domesticated t’ef accessions, plus three *Er. pilosa* accessions from Afghanistan (PI 223259, PI 221925, PI 211030) and the *Er. cilianensis* subsp. *staros* individuals (PI 211029, from Afghanistan). The second group included most *Er. pilosa* accessions (from Afghanistan, India, Iran, Pakistan and undetermined locations), individuals of *Er. cilianensis* (PI 197425, from Ethiopia) and one *Er. tenulifolia* accession (PI 213509, from India). The third group included one *Er. pilosa* (PI 271567, from India), the *Er. tremula* accessions (from Niger and Nigeria), one *Er. tenulifolia* from Ethiopia (PI 196852) and the undetermined *Eragrostis* species (PI 331389). For phylogenetic analysis, we computed the neighbour-joining (NJ) distance-based clustering method. Using the cophenetic function in adegenet, this method performed better than Unweighted Pair Group Method with Arithmetic Mean (UPGMA) or bioNJ. The phylogeny separated wild *Eragrostis* from t’ef but, similarly to PCA, clustered three *Er. pilosa* accessions and the *Er. cilianensis* subsp*. staros* with t’ef (figure 1). The Structure analysis based on the 4455 SNPs corroborated these results. The models with two (*K* = 2) and three (*K* = 3) gene pools were the most likely (figure 1C; electronic supplementary material, figure S2). *Eragrostis cilianensis, Er. tenulifolia* and *Er. tremula* are considered to derive from a different gene pool (shown in red) then the one from *Er. tef* descends (in grey), indicating the true wild status for the former. Within *Er. pilosa* accessions, the three Afghanistan accessions abovementioned (PI 223259, PI 221925 and PI 211030), plus the *Er. cilianensis* subsp. *staros*, are shown as completely sharing their gene pool with t’ef. However, some *Er. pilosa* accessions—PI 271567 from India, PI 442487 and PI 263510 (the latter two reported as originated from Belgium and the USA, meaning its original provenance is unknown)—clearly get their alleles from the same ancestral population as other wild *Eragrostis* species. The remaining *Er. pilosa* accessions get alleles from both the same gene pool as t’ef and the wild gene pool. *Eragrostis cilianensis* and *Er. pilosa* had the highest genetic diversity (measured as *H*_O_ and *H*_E_) whereas t’ef and *Er. cilianensis* subsp. *staros* had the lowest (table 1).

**Table 1.** Four measures of genetic diversity quantified for a panel of *Eragrostis* accessions based on DArTSeq and SilicoDArT markers and Structure-identified populations. (*H*_O_, observed heterozygosity; *H*_E_, expected heterozygosity.)

Species / Population	N	Ho	He	Allelic Richness	Private Alleles	Ho*	He*	Allelic Richness*	Private Alleles*
Complete Eragrostis accession Panel									
Er. cilianensis	3	0.89274993	0.44696227	1.536476	0	0.01717222	0.010950603	1.01217	0
Er. cilianensis subsp staros	3	0.04061708	0.02032308	1.024394	0	0.01242288	0.009017625	1.009699	0
Er. pilosa	14	0.53737143	0.39820141	1.403786	10	0.41461978	0.432373872	1.431896	512
Er. tef	64	0.04328047	0.02343002	1.023592	1293	0.02618644	0.035104108	1.035051	824
Er. tenuifolia	6	0.49138866	0.37377715	1.384629	0	0.20854058	0.256947273	1.253155	171
Er. tremula	4	0.04347227	0.02495955	1.028054	0	0.06430323	0.060059705	1.060005	0
Ethiopian t’ef accessions									
K = 3 Pop 1 (Purple)	5	3.01E−01	0.2397661	1.529009	53	0.9950447	0.4980336	1.951443	0
K = 3 Pop 2 (Black)	41	3.95E−01	0.3389388	1.731746	3441	0.9956371	0.4985218	1.88194	425
K = 3 Pop 3 (Green)	14	3.84E−01	0.3079326	1.664337	34	0.9888908	0.4981306	1.899967	0

^a^ Based on SilicoDArT markers.

**Figure 2.**
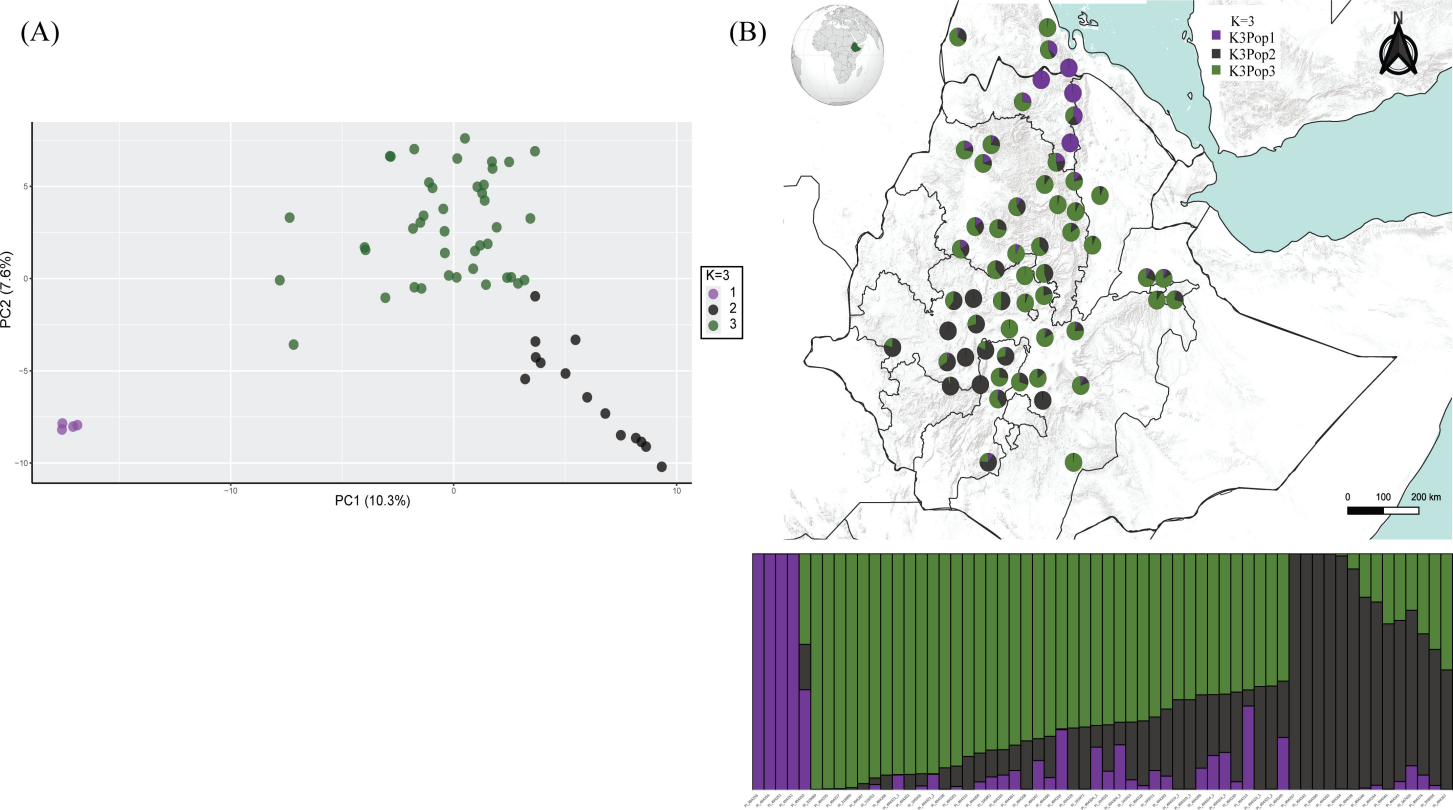
Population structure of 60 Ethiopian t’ef accessions, based on 5311 DArTSeq SNPs. (A) The first and second principal components of a PCA, with accessions coloured according to the cluster in the Structure *K* = 3 model from which they have they get most of their proportional membership. (B) Proportional memberships in the Structure *K* = 3 model with each accession represented in a geographical map as a pie chart in the location it was collected and with each slice indicating the proportional membership to each of the three groups.

We ran the same dataset but included only those accessions that were clustered in the above-mentioned first group (i.e. t’ef + three *Er*. *pilosa + Er. cilianensis* subsp. *staros*). This second PCA isolated the *Er. pilosa* accessions and, within the bulk of t’ef accessions, separated the Yemenite individuals (PI 618761), evidencing that these are genetically distinct from the Ethiopian accessions (electronic supplementary material, figure S3). We then prepared another dataset containing only the t’ef accessions from Ethiopia (*n* = 60) to assess fine population structure with PCA and Structure (5311 SNPs). The latter determined *K* = 3 as the most likely model, again corroborated by PCA (figure 2). A set of accessions restricted to the Tigrai region (dark purple in figure 2) constituted a distinct group whereas the remaining accessions formed two groups, distributed throughout Ethiopia (dark grey and dark green in figure 2). The Tigrai population had a lower genetic diversity compared with the other two populations in the *K* = 3 model (table 1).

The map in the electronic supplementary material, figure S4 shows the probability of finding the alleles that characterize t’ef populations based on extrapolation of complete panel Structure *K* = 3 Q-matrixes. It shows that this is a group of accessions restricted to the Tigrai region of the NHE. In figure 2, the same approach shows the geographical distribution of the three main populations of t’ef in Ethiopia. The restrictedness of population 1 to Tigrai is evidenced whereas the other two populations are spread throughout the land.

Population structure in crops is influenced by adaptation to climatic conditions and selective factors by communities in different environmental regimes [82–84]. To determine the interplay between geology, soil types and climate on the domestication process of *Er. tef*, we compiled the information and plotted these factors against the t’ef accessions. None of the categories for soil type, geological formation, or climate correlated with the population structure based on genome-wide SNP data (electronic supplementary material, figure S5).

### (b) Eleusine

**Figure 3.**
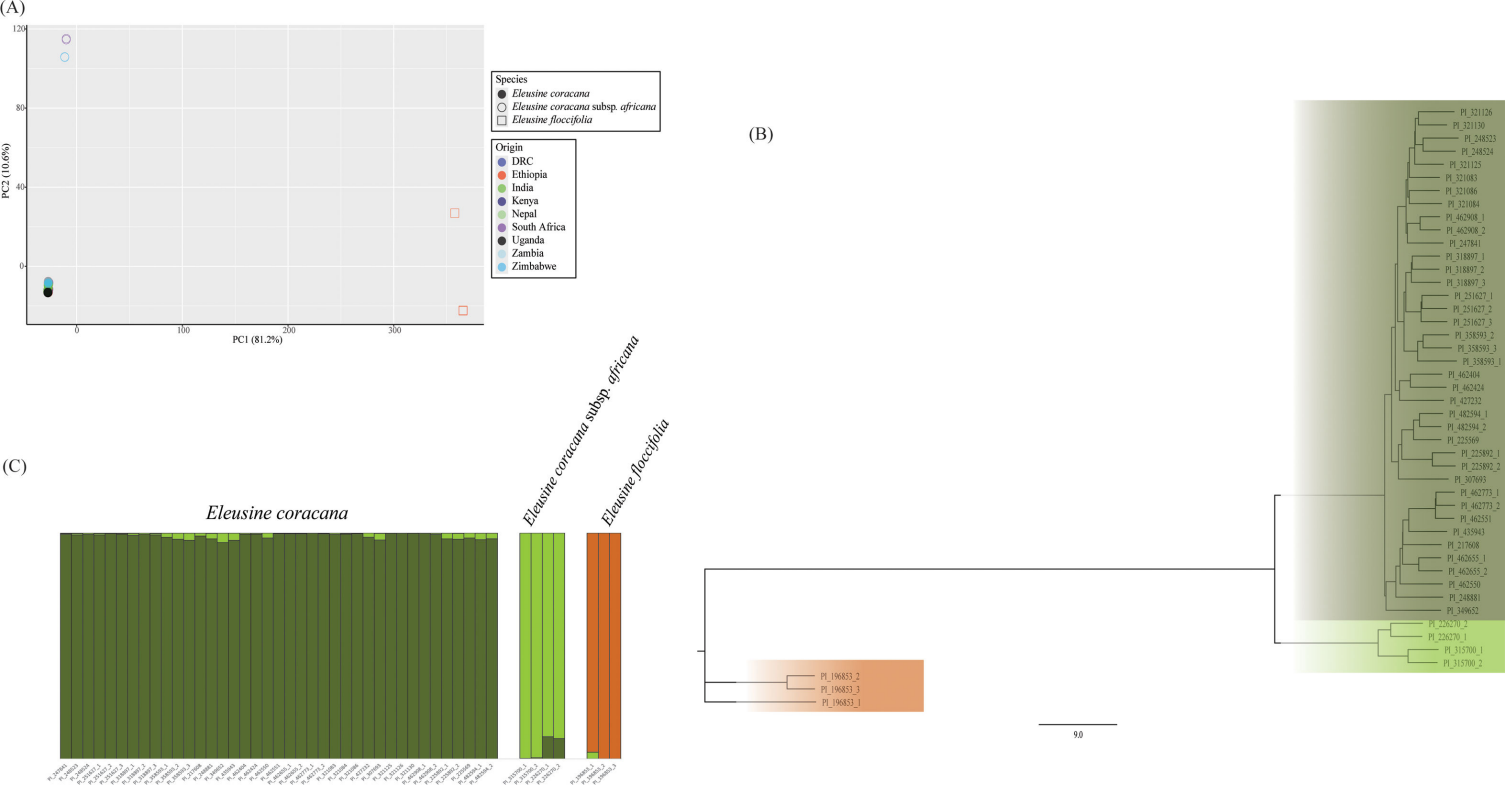
Population structure of 46 wild and domesticated *Eleusine* accessions, based on 5674 DArTSeq SNPs. (A) First and second principal components of a PCA. Points are coloured according to the taxonomical classification reported in the germplasm bank’s passport data and their country of origin. (B) NJ phylogenetic tree based on pairwise genetic distances calculated in the R package adagenet. (C) Graphical representation of a Q-matrix of the Structure *K* = 3 model.

The DarTSeq method uncovered 57 331 SNPs for the *Eleusine* set, from which (after quality filtering for coverage, MAF and missing data thresholds) 5674 SNPs were retained. The DArTSilico set contained 59 733 markers from which 2929 were kept after filtering. PCAs for both types of data were broadly coincident (figure 3A; electronic supplementary material, figure S6). The accessions were split into three distinct groups that, unlike t’ef, fitted their species classification. One included all the domesticated finger millet (*El. coracana*), the other the *El. coracana* subsp. *africana* and the third included only *El. floccifolia*. The phylogeny showed that *El. coracana* subsp. *africana* is evolutionary much closer to domesticated finger millet than *El. floccifolia* (figure 3B). Structure also confirmed this observation. The model with three (*K* = 3) gene pools was the most likely, and in this the three species are clearly distinct (figure 3C). However, some finger millet accessions share a small amount of alleles with the *El. coracana* subsp. *africana* gene pool whereas no such common genetic ancestry occurs with *El. floccifolia.* The highest genetic diversity (measured as *H*_O_ and *H*_E_) was observed in *El. coracana* subsp. *africana*, twice the diversity of *El. floccifolia* and the lowest in finger millet (table 2).

**Table 2.** Four measures of genetic diversity quantified for a panel of *Eleusine* accessions based on DArTSeq and SilicoDArT markers and Structure-identified populations. (*H*_O_, observed heterozygosity; *H*_E_, expected heterozygosity.)

species/population	*n*	*H*_O_	*H*_E_	allelic richness	private alleles	*H*_O_ ^a^	*H*_E_ ^a^	allelic richness^a^	private alleles^a^
	complete *Eleusine* accession panel								
*Eleusine coracana*	39	0.0228065	0.02533213	1.025303	31182	0.5998786	0.3219945	1.325766	16946
*Eleusine coracana* subsp*. africana*	4	0.07086417	0.0450162	1.048624	786	0.7523045	0.38423353	1.438528	0
*Eleusine floccifolia*	3	0.02743285	0.02662777	1.025955	23655	0.1655855	0.08159185	1.101605	0
	cultivated finger millet accessions								
*K* = 3 pop 1 (red)	22	0.1461406	0.1781129	1.512621	2224	0.7161132	0.3743538	1.794716	381
*K* = 3 pop 2 (orange)	6	0.2199941	0.2188531	1.513276	1431	0.7633245	0.3971454	1.828333	186
*K* = 3 pop 3 (blue)	11	0.1489139	0.196185	1.486129	2221	0.7771028	0.4078002	1.843455	573

^a^ Based on SilicoDArT markers.

**Figure 4.**
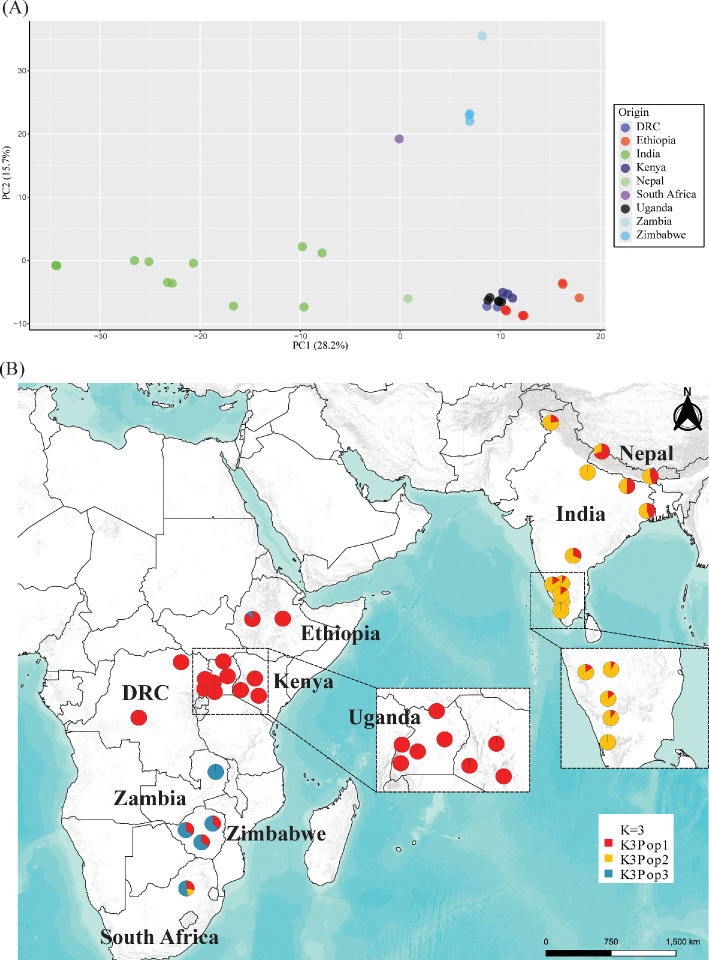
Population structure of 39 cultivated finger millet accessions, based on 2044 DArTSeq SNPs. (A) First and second principal components of a PCA, with accessions coloured according to their provenance. (B) Proportional memberships in the Structure *K* = 3 model with each accession represented in a geographical map as a pie chart in the location it was collected and with each slice indicating the proportional membership to each of the three groups.

We ran the same dataset but included only finger millet accessions (*n* = 39). This produced a dataset with 2044 SNPs, after quality filtering, and 1097 markers for the DArTSilico set. The PCA separated African from Asian (India and Nepal) accessions along the PC1 axis and East Africa and Central Africa (Ethiopia, Kenya, Democratic Republic of Congo and Uganda) from South African (Zambia, Zimbabwe and South Africa) accessions (figure 4A). The Structure models *K* = 2, *K* = 3 and *K* = 5 had the highest likelihood, with the former showing a separation between the African and Asian gene pools (electronic supplementary material, figure S7 red and orange, respectively,). In the *K* = 3 model, the African gene pool gets separated into two sub-populations: one that includes the East and Central African accessions (Ethiopia, Kenya, Uganda and the DRC) and another encompassing the southern African ones (Zambia, Zimbabwe and South Africa). The *K* = 5 model separates the Ethiopian accessions from the rest of the East African ones and detects two Asian sub-populations (figure 4B). The accession from South Africa, interestingly, gets most of its alleles from this south African sub-population but also has a high proportion of alleles from the Asian populations. Populations and sub-populations that group accessions from Africa (population 1 in the *K* = 2 model) have slightly higher genetic diversity (*H*_O_ and *H*_E_) than those that include Asian ones (table 2), and within the former, southern African ones (in the *K* = 3 model) have higher genetic diversity than the East African ones.

## 4. Discussion

DArTSeq proved to be an effective and cheap way to investigate the domestication of these two non-model African crops from a genomics point on view. The number of high-quality filtered SNPs, both based on reads or on presence–absence of enzyme cutting (SilicoDArT) was in the 1000s, which is high enough to determine population structure and the geographical distribution of genetic diversity from which history and adaptation can be inferred. The allotetraploid genome of t’ef and finger millet introduces challenges for SNP discovery owing to the presence of homoeologous chromosomes from different sub-genomes. This can lead to errors such as false-positive SNPs and ambiguous read mapping. To address these, specific analytical treatments were used namely subgenome-specific SNP calling [85]. The proprietary variant calling algorithm of DArTSeq is ploidy-aware and accounts for multiple alleles at each locus, improving SNP accuracy in polyploids [86] taking advantage of read mapping and filtering techniques based on read depth and allele frequency to minimize ambiguity and ensure reliable SNP detection. T’ef and finger millet were analysed here side-by-side owing to their shared characteristics and their importance as crops adapted to dry conditions in Africa. Both species belong to the Chloridoideae subfamily of Poaceae, which is highly adapted to arid environments. We considered these species together because they are local, understudied in comparison with other crops and, mostly, because archaeobotanical data available for both species suggests they were both part of early agriculture in the NHE. We hypothesize these two crops share similar evolutionary histories. The main issue was the limited number of accessions available for distribution in germplasm banks suited for this type of study, namely t’ef accessions from outside Ethiopia (Yemen was the sole exception) and wild forms of both t’ef and finger millet from the Horn of Africa, where archaeobotanical evidence suggests that domestication occurred. Our results also suggest that mislabelling of accessions, erroneous passport curation or ferality pose problems that need to be addressed. The data generated, however, can be useful for crop breeding and for further studies on crop adaptation that were beyond the scope of this article (electronic supplementary material, S3−S6).

### (a) Eragrostis

The clustering together of all t’ef accessions, including the Yemen ones, argues for a single domestication (figure 1). It is only when wild plants are removed from the analysis that the Yemenite t’ef reveals its genetic distinctiveness, probably owing to an early introduction and evolution in separation from other t’ef varieties from elsewhere that could introduce novel alleles (electronic supplementary material, figure S3). It is noteworthy that all clustering methods used included in the group of domesticated t’ef, three wild *Er. pilosa* and the *Er. cilianensis* subsp*. staros* accessions, all from Afghanistan. This observation can be explained by: (i) accessions were misclassified by germplasm banks; (ii) an origin of t’ef in Afghanistan; (iii) these accessions descend from an ancestral population of *Er. pilosa* that extended to the NHE from which t’ef was also domesticated; and (iv) these wild accessions are not wild at all, but feral forms of t’ef previously cultivated in these regions that were erroneously classified as wild species. The first hypothesis can apply to *Er. cilianensis* subsp*. staros,* as in all clustering methods these accessions are indistinguishable from t’ef. However, the three *Er. pilosa* accessions genetically closer to t’ef were consistently separated when all these accessions are analysed together (electronic supplementary material, figure S3). The second hypothesis would imply that t’ef emerged in Central Asia and was brought to the NHE in the second millennium BCE, which is not supported by any archaeology and previous botanical studies [87]. To test the third hypothesis, it would be necessary to conduct genetical analysis on *Er. pilosa* accessions from Ethiopia or surrounding areas (we could not find any available in germplasm banks) and apply methods that separate introgression from common descent like ABBA-BABA tests [88]. The former was attempted in an amplified fragment length polymorphism study by Ayele *et al*. [89], which included three Ethiopian *Er. pilosa* and all the *Er. pilosa* we tested in this study. Their results were like ours, with the three Afghanistan *Er. pilosa* accessions (plus two Ethiopian) clustering with t’ef and the other *Er. pilosa* (including one Ethiopian) being separated. Again, this suggests that t’ef descends from a particular *Er. pilosa* population that extended in the past from the Horn of Africa to Central Asia. The fact that in the Structure analysis, four *Er. pilosa* accessions from Afghanistan, Pakistan and Iran share around a third of their alleles with the domesticated t’ef population and three others get most of their alleles from the t’ef population could indicate that. Alternatively, the fourth hypothesis proposes that species classified as *Er. pilosa* by botanists on the basis of morphological criteria might include feral forms of t’ef, or at least plants that have been intensively introgressed by alleles derived from cultivated t’ef plants. This confusion between wild and feral forms has indeed been detected in other crops such as eggplant [90], rice [91], barley [92], turnip [93], sorghum [94] or pearl millet [95]. To us this seems the more parsimonious explanation.

It is also possible that t’ef was domesticated from a species not included in this study and, in fact, various other species have been proposed as putative progenitor, including *Eragrostis aethiopica, Eragrostis lugens, Eragrostis obtus*a or *Eragrostis ferruginea* [96,97]. Molecular studies based on nuclear genes and chloroplast genomes do point *Er. pilosa* as the species closest to domesticated t’ef [34,98]. However, these studies were based on a few accessions of *Er. pilosa*. For example Ingram & Doyle [34], screened only four accessions of this wild taxon, from Afghanistan, Pakistan, India and Iran. In their study using genotype-by-sequencing derived nuclear SNPs from 40 accessions of wild *Eragrostis* species, Girma *et al*. [97] included only one accession of *Er. pilosa* with a provenance from France. Moreover, *Er. lugens*, one of the species identified by their Structure results to belong to the same gene pool as t’ef, was represented as a single accession from Uruguay; considering that *Er. lugens* is typically a South American species it is unlikely to be related to t’ef. Our results point in the direction that some *Er. pilosa* accessions may in fact be feral whereas others from the same regions are *bona fide* wild forms (p.ex. accession PI 271567 in our panel). The sampling of feral rather than wild forms might confuse phylogenies. In our case, excluding those three accessions from Afghanistan (PI 223259, PI 221925 and PI 211030) that are clustered with domesticated t’ef, *Er. pilosa* accessions are not shown in the PCA as genetically closer to t’ef than any of the other taxa sampled. It is possible that the wild progenitor of t’ef is not yet identified or that t’ef does not have a wild progenitor having simply been cultivated intensively since the second millennium BCE and then introduced in other parts of the Old World. However, the low genetic diversity (table 1) observed compared with other wild species is typical of crops that were domesticated from a restricted population of wild progenitor species [99].

**Figure 5.**
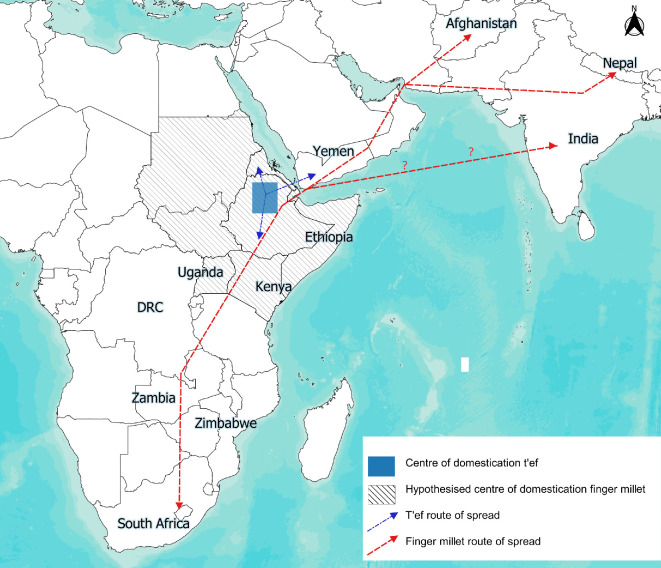
Proposed routes of dispersal for t’ef and finger millet from their core area of domestication based on the geographical distribution of present-day genetic diversity.

The fact that the population structure in Ethiopian t’ef could not be explained by soil type, administrative region, climate zone, Köppen climate classification or local geology (electronic supplementary material, figure S5) suggests that these structures do not reflect adaptation, although other types of tests might be needed to confirm this. Southwestern Ethiopia is recognized as the domestication centre of crops such as *Co. arabica* and *En. ventricosum* [20,100–103]. It has been proposed that t’ef might have been domesticated there [100]. However, archaeological evidence in southwestern Ethiopia suggests the later emergence there of ceramic technology and animal food production (500–1000 CE) [104–108]. We propose that this structure is explained by the historical dynamics of people moving from the NHE to southwestern Ethiopia and bringing their crop seeds with them. Population movements southward from the northern highlands followed the weakening of the Aksumite empire during the late first millennium CE [109]. This could have facilitated crop dispersal (figure 5). T’ef chaff from Lalibela cave near Lake Tana, dated to around the twelfth century CE [110,111], supports this notion. However, the limited research conducted in the central and southern regions of Ethiopia poses a significant obstacle to fully understanding the domestication route of t’ef.

The distinctiveness of the Tigrai cluster (population 1) can be explained by the crop being domesticated elsewhere and introduced recently. Alternative explanations are that it reflects an isolated farming community that has not received germplasm from other regions; the selective forces of adaptation to high altitude; or that this is indeed a relic population that is closer to the original domesticated t’ef. We favour the latter as it fits the currently available archaeobotanical evidence. The earliest evidence of t’ef cultivation originates from the NHE, as evidenced by archaeological, phytolith and starch analyses at the Mezber site dating back to 1600 BCE [29,50,51]. Further evidence comes from the present-day Eritrean site Mai Chiot dated to 300−400 BCE [112]. It is likely that t’ef was domesticated in the NHE in the second millennium BCE and only later, in Aksumite times, a small number of genotypes were brought from this core region and introduced to farming communities throughout the country, creating a genetic bottleneck that explains the genetic distinctiveness. The geographical distribution of the two other populations in the model *K* = 3 suggests that two major spreads from the core area might have occurred, either from distinctive groups within the NHE or at different time periods. However, the lower genetic diversity of the Tigrai t’ef, compared with the other two groups, suggests a more recent introduction (in which case this would not be an ancestral population). Alternatively, a reduction in the acreage devoted to this relic population can also lower overall genetic diversity.

The genetic difference of the Yemenite accessions points to an ancient separation from the NHE gene pool. T’ef has been found at the first millennium BCE site of Hajar bin Humeid, indicating an early spread of this crop across the Red Sea [113]. It is likely that no new introductions were made since that early period, allowing the Yemenite population to diverge genetically. To our knowledge, t’ef has not been reported in Harappan sites or in the Indian archaeobotanical record, except for the presence of phytoliths of Chloridoid grasses (*Eragrostis* or *Cynodon*) interpreted as weeds of rice fields [114]. So, it is likely that t’ef cultivation never spread beyond the Horn of Africa, surrounding areas of the Arabian Peninsula and—considering our genetic data and assuming accessions were not mislabelled —Afghanistan.

### (b) Eleusine

The population structure analysis of *Eleusine* shows a much more clear-cut apportioning by taxonomy than *Eragrostis*. No potential feral/misclassified accession was detected in our data. This contrasts with previous work on wild and domesticated finger millet, which found evidence of some hybrid accession and at least nine accessions labelled as *El. coracana* subsp. *africana* that were in fact finger millet [45,115]. It is noteworthy that both species are tetraploid and self-pollinating, so the occurrence of gene flow between populations is expected to be minimal, and this is indeed the case with *Eleusine*. This might also be because accessions of the wild progenitor *El. coracana* subsp. *africana* were from South Africa and Zimbabwe, not from East Africa, around where finger millet is assumed to have been domesticated. It is possible that the clear genetic distinctiveness between *El. coracana* subsp. *africana* and finger millet could be owing to the latter descending from an East African population of *El. coracana* subsp. *africana* that was genetically quite distinct from the South African populations of *El. coracana* subsp. *africana* sampled here. Unfortunately, seed banks had no available wild and domesticated finger millet accessions from Ethiopia that would allow a closer comparison between the crop and its putative wild progenitor population. According to a study [116], this wild species might not be found in Ethiopia or neighbouring countries, only the occasional feral form or populations derived from the hybridization with another wild species *Eleusine kigeziensis*. In June 2024, a query on the Genesys database of the Plant Genetic Resources for Food and Agriculture (https://www.genesys-pgr.org/) did not retrieve any *El. coracana* subsp. *africana* accession from Ethiopia or the Horn, although some are reported in the Global Biodiversity Information Facility, all prior to 1974 (https://www.gbif.org/) and some accessions from this area are mentioned in more recent molecular work [45,115]. It might be the case that in the past, there were wild populations of *El. coracana* subsp. *africana* in Ethiopia that disappeared before twentieth century botanists could sample them and most accessions observed today are feral forms. In any case, these are difficult to access. Liu *et al*. [116] studied the phylogeny of the genus *Eleusine* place sets of finger millet and *El. coracana* subsp. *africana* together in different clades, which might be interpreted as evidence for multiple domestication events. Our data do not support this, as both in phylogenies and PCA/Structure analysis, all the domesticated finger millet are separated from all the wild *El. coracana* subsp. *africana*. The much lower genetic diversity of the cultivated accessions points towards a single domestication event from a single (or small number of nearby) wild population. One possibility is that finger millet might have been domesticated south of the Horn (in present-day Kenya or Uganda) and later fully adopted as a crop in the NHE, where the earliest archaeobotanical records of this species appear, at the sites of Ona Nagast, D-site, K-site, Mezber and Ona Adi [51,112,117,118]. To validate this hypothesis, it would be necessary to find remains of finger millet in East Africa south of the Horn contemporary or older than the remains found in the NHE. The oldest securely dated finger millet remains found outside Ethiopia; however, are from the Kakapel rock shelter in western Kenya, dated to *ca* 700−900 CE [119]; the Mgombani and Panga ya Saidi sites, in Kenya, from 666−890 cal. CE [120]; Gogo Falls, Kenya, dating to the first−second millennia CE (121); Deloraine site in Kenya, 800 CE [[122]], the Munsa site in Uganda, also dated to the late Iron Age [123]; and the sites of Karama and Musanze in Rwanda, between 750 and 1150 CE [124]. There is also a report of finger millet in the Nok culture site of Ungwar Kura in Nigeria, incongruously dated to 400−200 cal. BCE, although these are isolated finds [125]. To be sure, the oldest finger millet seed remains in Ethiopia occur between 50 BCE and 700 CE, at the above-mentioned sites, with earlier (first millennium BCE) occurrences being only of phytoliths that cannot be distinguished from other grasses, including wild species [126]. Taken together, it is not impossible that this crop originated in the Great Lakes region of East Africa and was introduced in the NHE through the movements of people. The hypothesis of a NHE origin could also be investigated by the genetic analysis of wild populations in Ethiopia and by finding if these are genetically closer to the set of domesticated finger millet than to the wild populations of East Africa.

It is clear, however, that *contra* what early authors such as Vavilov and de Candolle hypothesized, finger millet was not independently domesticated in India [44]. This hypothesis gained some traction as some finger millet archaeological grains found in Harappan sites in the Indian sub-continent were dated to the second millennium BCE, so much earlier than the first evidence of this crop in Africa [127,128]. These included Oriyo Timbo, Babar Kot, Hulas, Shikarpur and Surkotada [129]. Because these were found alongside remains of sorghum, an undisputed African domesticate, it was proposed that instead of domestication in the Indian sub-continent, finger millet could have been domesticated in the Sahel region, where sorghum and pearl millet originated, and introduced in India at an early stage through a maritime route [130,131]. This, however, has been questioned, with researchers arguing that the Harappan finger millet remains were most likely misidentified grains of *Setaria*/*Brachiaria* [132–135]. In our data, all finger millet accessions tested, whether from Africa or India/Nepal, are clustered together and show high genetic similarity, indicating a single introduction of this crop to have occurred at a distant enough past for divergence between gene pools to occur. Determining a divergence date based on DArtSeq genetic data is, currently, not recommended, though. DArTSeq is primarily designed for high-throughput SNP discovery and genome-wide diversity analysis, focusing on identifying genetic variation across individuals or populations. However, DArTSeq typically targets anonymous loci scattered across the genome, which are not always linked to conserved regions necessary for calibrating molecular clocks and estimating divergence times [73]. By contrast, methods like RADSeq or full-genome sequencing often target specific loci or regions where mutation rates can be reliably calculated and used for evolutionary dating [136]. The anonymous nature of DArTSeq loci means that while it is highly effective for population genomic studies, association mapping and diversity assessments, it is less suitable for applications that require precise evolutionary timing [137]. This is owing to the lack of control over the evolutionary rates of the regions being sampled, which is crucial for molecular dating.

Linguistic evidence suggests that the spread of finger millet to Asia may have occurred in classical antiquity, as the Asian names for the crop have no resemblance to the diversity of names used in African languages [138]. Our genomic data confirm this separation, as different regions form distinct genetic groups, and one of them includes only Asian accessions (figure 4). This suggests two routes out of the main domestication centre: (i) eastwards, across the Red Sea and the Arabian Peninsula or the Indian Ocean [139]; and (ii) southwards from Ethiopia through Uganda and Kenya, ultimately reaching southern Africa (figure 5). The archaeobotanical record in the coastal parts of the Arabian Peninsula is lacking, and the few data available do not indicate the presence of finger millet until late medieval times [140–142]. On the other hand, long-distance trade across the Indian Ocean is well established by the first millennium BCE and probably even before [143]. Until more archaeobotanical work is carried out on the southern shores of the Arabian Peninsula and early agriculture there is established, a land route from the NHE to the Indian sub-continent should not be excluded.

A southward movement of finger millet was initially proposed by Ehret [144], whose linguistic analysis suggested that this crop originated in the NHE and gradually dispersed to the south with Cushitic-speaking groups. The slightly higher diversity of African accessions compared with Asian ones (table 2) is to be expected since it was in Africa that finger millet was domesticated. However, it is interesting that southern African accessions have a slightly higher diversity than East African ones, as its cultivation in southern Africa is more recent. The emergence and spread of pastoralism and farming in southern Africa dates around the first millennium CE [145], and the finger millet’s arrival there was probably associated with the practise of a mixed economy by these groups, supplementing pastoralism with small-scale farming. This higher genetic diversity in finger millet may reflect less intensive selection in southern Africa, where finger millet tends to be a secondary crop. Interestingly, the accession from South Africa has an ancestry from both the southern African and Indian gene pools. This could result from an admixture between native finger millet and accessions brought from India during British colonialism. This mixing of varieties from different continents could explain the higher diversity. It also leads to considering back-to-Africa events and recent crop movements when investigating the history of these crops.

### (c) Domestication and spread

When we compare these two crops, some differences are observable despite the similar biology (i.e. tetraploidy, self-pollinating habit and belonging to the same sub-family). Genetic diversity in finger millet shows a broad geographical distribution that fits regional groups (i.e. East Africa, southern Africa, Indian sub-continent); the domesticated are clearly separated from wild accessions; and accessions from the proposed centre of origin, i.e. the highland region from Ethiopia to Uganda, [46], make up a single population. By contrast, with t’ef, genetic similarities are found between domesticated forms from Ethiopia and accessions classified as wild from afar regions like Afghanistan; some wild accessions (*Er. pilosa*) cluster with domesticated forms, whereas others do not; and in Ethiopia a genetically distinct population occurs in the Tigrai region, suggesting a more dynamic crop evolution. Although both crops were probably domesticated in the NHE or surrounding regions, their spread was considerably different. For example, t’ef never spread into East and southern Africa, only across the Red Sea into Asia. This suggests that agricultural spread in Africa did not include a crop package, but the selective adoption of some species and the rejection of others. For example, although southwest Asian domesticates like wheat and barley were widely cultivated in the NHE, these did not spread to other parts of Ethiopia until the mid-first millennium CE and only reached southern Africa during the seventeenth century onwards. This selective adoption of some crops over others is seen in other contexts. Foxtail and broomcorn millets were domesticated in China around 6000−5500 BCE, and by the second millennium BCE, they had been adopted in Europe as far away as the Iberian Peninsula [146,147]. However, the most important Asian domesticate, rice, was not cultivated in Europe until the Middle Ages. Likewise, southwest Asian cereals (wheat and barley) were adopted in China by the second millennium BCE, but none of the pulses is known to have travelled east [148]. In India, a series of crops were adopted from both west and east Eurasia and mixed with local domesticates, using crop diversity to promote resilience [149–151]. Within Africa, some crops were also selectively adopted in different regions. Sorghum and pearl millet were somehow cosmopolitan in the Sahel and parts of East Africa, but African rice, another crop from the Sahel, was not widely adopted [152]. The reasons why some crops were adopted and others rejected throughout African prehistory are unclear and can include: cultural preferences for taste, texture or shape, adequacy to pre-existing culinary practises, suitability of certain crops to particular soil and environments, value attributed to certain foods by elites or commoners, integration (or not) of novel crops in established farming systems, interplay with pastoralism and availability of contact networks through which crops can be introduced.

The possibility that t’ef and finger millet domestication were a response to environmental change has rarely been addressed [112]. There were periods of climatic aridity and humid phases in the Horn of Africa [153–155]. These could have promoted more investment in the cultivation of well-adapted local plants as a response to drought or, with climate amelioration, the experimentation of novel crops. During the period of the earliest evidence for t’ef domestication, around 1600 BCE, the climate changed to a drier phase [153,156–161]. It could have been during this period that a phase of intensive cultivation occurred in the NHE, and then spreading as fully domesticated crops to neighbouring regions during the later climatically stable phase that characterized the Aksumite period. The genomic data presented here could be mined to find alleles fixed in the domesticated forms and absent (or infrequent) in the wild forms [162]. If such alleles are found to be associated with genes involved in environmental responses, the case for climate change as a driver of t’ef and finger millet domestication would be reinforced.

Obtaining a complete picture of the domestication process of both t’ef and finger millet is hindered by biases in the archaeobotanical record [119]. These include a skew towards regions where excavation was carried out using floatation methods (e.g. this has been performed more systematically in Tigrai region of Ethiopia than in other eastern Africa regions with few exceptions like [163]), taphonomical issues and adverse conditions towards the preservation of plant remains in many parts of the regions of interest [123]. The relative lack of interest of archaeologists in plant domestication in the Horn and eastern Africa, regions where research into the Early Stone Age is much more intensive, is also to be considered. Another issue is dating the domestication events. T’ef and finger millet have very small grains that can easily experience bioturbation and sink along site profiles from more recent layers [164]. In addition, the preparation of these crops into food does not often involve roasting, meaning finding charred remains is difficult and even if it is charred preservation is very minimal [112].

In conclusion, it would be ideal to revisit finds of seed remains from these regions and date them directly using the accelerator mass spectrometry method [165]. This would improve the chronology of domestication events. Using genomics of present-day crop landraces and wild plants is also plagued by biases in the availability of plants collected and stored by seed banks and by the extinction dynamics of populations of wild progenitors or species, owing besides other reasons, to changes in land use. However, when a large panel of accessions is available, DArTSeq is a cheap and effective method to discover genetic variants between crops from their wild progenitors and, within the former, between distinct populations that reflect past events such as the spread of cultivation, adaptation to different environments and cultural preferences. To sum up, the current research exemplifies how modern genetic analysis can contribute to elucidate the origins of food production. Additionally, interdisciplinary research integrating genetics, archaeology and environmental studies is imperative to uncover the precise domestication routes and evolutionary history of significant crop species. By adopting a multidisciplinary approach to address existing biases and gaps in the different fields, we can enhance our understanding of the origins of agricultural practises in Africa.

## Data Availability

All data are available in the manuscript or the electronic supplementary material [166].

## References

[1] Childe VG. 1952 New light on the most ancient east., 4th edn. London, UK: Routledge & K. Paul.

[2] Smith BD. 2001 The transition to food production. In Archaeology at the millennium: a sourcebook (eds GM Feinman, TD Price), pp. 199–229. New York, NY, USA: Kluwer Academic/Plenum Publishers.

[3] Smith BD. 2001 Documenting plant domestication: the consilience of biological and archaeological approaches. Proc. Natl Acad. Sci. USA **98**, 1324–1326. (10.1073/pnas.98.4.1324) 11171946 PMC33375

[4] Barbier EB. 2010 Scarcity and frontiers: how economies have developed through natural resource exploitation. Cambridge, UK: Cambridge University Press.

[5] Herrera RJ, Garcia-Bertrand R. 2018 The agricultural revolutions, pp. 475–509. Amsterdam, The Netherlands: Elsevier Science & Technology.

[6] Vavilov NI. 1926 Centers of origin of cultivated plants. Bull. Appl. Bot. Genet. Plant Breed. **13**, 1–64.

[7] Vavilov NI. 1951 The origin, variation, immunity and breeding of cultivated plants. Soil Sci. **72**, 482. (10.1097/00010694-195112000-00018)

[8] Harlan JR. 1971 Agricultural origins: centers and noncenters. Sci. Am. Assoc. Adv. Sci. **174**, 468–474. (10.1126/science.174.4008.468) 17745730

[9] Harlan JR. 1969 Ethiopia: a center of diversity. Econ. Bot. **23**, 309–314. (10.1007/bf02860676)

[10] Murdock GP. 1959 Africa: its peoples and their culture history. New York, NY: McGraw-Hill.

[11] Clark JD. 1976 Germany: prehistoric populations and pressures favoring plant domestication in Africa. In Origins of African plant domestication (ed. JR Harlan), pp. 67–106. Germany: De Gruyter Mouton Inc. (10.1515/9783110806373.67)

[12] Doggett H. 1991 Sorghum history in relation to Ethiopia. In Plant genetic resources of ethiopia (eds JMM Engels, JG Hawkes, M Worede), pp. 140–159. Cambridge, UK: Cambridge University Press. (10.1017/CBO9780511551543.011)

[13] Doggett H. 1988 Sorghum history in relation to Ethiopia (ed. JMM Engels). In The Conservation and Utilization of Ethiopian Germplasm Proc. of an Int. Symp. October 13-16, pp. 97–115. International Board for Plant Genetic Resources (IBPGR): Addis Ababa, Ethiopia.

[14] Purseglove JW. 1976 The origins and migration of crops in tropical Africa. In Origins of african plant domestication (eds JR Harlan, JMJ Wet, ABL Stemler), pp. 291–310. The Hague, The Netherlands: Mouton. (10.1515/9783110806373.291)

[15] Stiehler W. 1948 Studien zur landwirtschafts- und Siedlungsgeographie Äthiopiens. Erdkunde **2**, 257–282. (10.3112/erdkunde.1948.02.07)

[16] Seligman CG. 1873 Races of Africa. (Home university library of modern knowledge; 144), 3rd edn. London, UK: Oxford University Press.

[17] Curtis M. 2005 Archeological investigations in the greater Asmara area: a regional approach in the central highlands of Eritrea. PhD thesis, University of Florida, Gainesville, FL, USA.

[18] Negash A. 2001 The Holocene prehistoric archaeology of the Temben region, northern Ethiopia. PhD thesis, University of Florida, Gainesville, FL, USA.

[19] Negash A, Marshall F. 2021 Early hunters and herders of northern Ethiopia: the fauna from Danei Kawlos. SINET **44**, 251–222. (10.4314/sinet.v44i2.8)

[20] Brandt SA. 1982 A late Quaternary cultural/environmental sequence from Lake Besaka, southern Afar, Ethiopia. PhD thesis, University of California, Berkeley, CA, USA

[21] Phillipson DW. 1977 The excavation of Gobedra rock-shelter, axum: an early occurrence of cultivated finger millet in northern Ethiopia. Azania **12**, 53–82. (10.1080/00672707709511248)

[22] Clark JD, Prince GR. 1978 Use-wear on later stone age microliths from laga oda, haraghi, Ethiopia and possible functional interpretations. Azania **13**, 101–110. (10.1080/00672707809511633)

[23] Woldekiros HS, D’Andrea AC. 2022 Complex (multispecies) livestock keeping: highland agricultural strategy in the northern horn of Africa during the pre-Aksumite (1600 BCE–400 BCE) and Aksumite (400 BCE–CE 800) periods. Front. Ecol. Evol **10**, 1–18. (10.3389/fevo.2022.901446)

[24] Khalidi L *et al*. 2020 9000 years of human lakeside adaptation in the Ethiopian Afar: fisher-foragers and the first pastoralists in the Lake Abhe basin during the African humid period. Quat. Sci. Rev. **243**, 106459. (10.1016/j.quascirev.2020.106459)

[25] Girma H. 2001 The emergence of prehistoric pastoralism in southern Ethiopia. PhD thesis, University of Florida, Gainesville, FL, USA.

[26] Fattovich R. 1990 Remarks on the pre-aksumite period in Northern Ethiopia. J. Ethiop. Stud. **23**, 1–33.

[27] Phillipson DW. 1998 Ancient Ethiopia: Aksum: its antecedents and successors. London, UK: British Museum Press.

[28] Phillipson DW. 2000 Archaeology at Aksum, Ethiopia, 1993-7. (Memoirs of the British Institute in Eastern Africa; no. 17). London, UK: British Institute in Eastern Africa ; the Society of Antiquaries of London.

[29] D’Andrea AC *et al*. 2023 The Pre-Aksumite Period: indigenous origins and development in the Horn of Africa. Azania **58**, 329–392. (10.1080/0067270x.2023.2236484)

[30] Harlan JR. 1982 The origins of indigenous African agriculture., pp. 624–657. Cambridge, UK: Cambridge University Press.

[31] Harlan JR. 1992 Agricultural origins in world perspective. In Indigenous African agriculture (eds PG Watson, CW Cowan), pp. 59–69. Washington, DC: Smithsonian Institution Publications in Anthropology.

[32] Harlan JR. 1992 Crops & man, 2nd edn. Madison, WI: American Society of Agronomy. (10.2135/1992.cropsandman)

[33] Bard KA, Coltorti M, DiBlasi MC, Dramis F, Fattovich R. 2000 The environmental history of Tigray (northern Ethiopia) in the middle and late Holocene: a preliminary outline. Afr. Archaeol. Rev. **17**, 65–86. (10.1023/A:1006630609041)

[34] Ingram AL, Doyle JJ. 2003 The origin and evolution of Eragrostis tef (Poaceae) and related polyploids: evidence from nuclear waxy and plastid rps16. Am. J. Bot. **90**, 116–122. (10.3732/ajb.90.1.116) 21659086

[35] Assefa K *et al*. 2015 Genetic diversity in tef [Eragrostis tef (Zucc.) Trotter]. Front. Plant Sci. **6**, 177. (10.3389/fpls.2015.00177) 25859251 PMC4374454

[36] Jones BMG, Ponti J, Tavassoli A, Dixon PA. 1978 Relationships of the Ethiopian cereal T′ef (Eragrostis tef (Zucc.) Trotter): evidence from morphology and chromosome number. Ann. Bot **42**, 1369–1373. (10.1093/oxfordjournals.aob.a085583)

[37] Tefera H, Ketema S, Tesemma T. Variability, heritability and genetic advance in tef (Eragrostis tef (Zucc.) Trotter) cultivars. Trop. Agric. **67**, 317–320. https://journals.sta.uwi.edu/ojs/index.php/ta/article/view/1825

[38] Tadesse D. 1993 Study on genetic variation of landraces of teff (Eragrostis tef (Zucc.) Trotter) in Ethiopia. Genet. Resour. Crop Evol. **40**, 101–104. (10.1007/bf00052640)

[39] Hundera F, Arumuganathan K, Baenziger P. 2000 Determination of relative nuclear DNA content of tef [Eragrostis tef (Zucc.) Trotter] using flow cytometry. J. Genet Breed **54**, 165–168.

[40] Bai G, Ayele M, Tefera H, Nguyen HT. 2000 Genetic diversity in tef [Eragrostis tef (Zucc.) Trotter] and its relatives as revealed by random amplified polymorphic DNAs. Euphytica. **112**, 15–22. (10.1023/A:1003802207158)

[41] Assefa K, Chanyalew S, Tadele Z. 2017 Tef, *Eragrostis tef* (Zucc.) Trotter. In Millets and sorghum, 1st edn (ed. JV Patil), pp. 226–266. Hoboken, USA: Wiley. (10.1002/9781119130765)

[42] Ganapathy KN. 2017 Improvement in finger millet: status and future prospects. In Millets and sorghum, 1st edn (ed. JV Patil), pp. 87–111. Hoboken, USA: Wiley. (10.1002/9781119130765)

[43] Hilu KW, De Wet JMJ, Harlan JR. 1979 Archaeobotanical studies of Eleusine coracana ssp. coracana (finger millet). Am. J. Bot. **66**, 330–333. (10.1002/j.1537-2197.1979.tb06231.x)

[44] Hilu KW, de Wet JMJ. 1976 Domestication of Eleusine coracana. Econ. Bot. **30**, 199–208. (10.1007/BF02909728)

[45] Dida MM, Wanyera N, Harrison Dunn ML, Bennetzen JL, Devos KM. 2008 Population structure and diversity in finger millet (Eleusine coracana) germplasm. Trop. Plant Biol. **1**, 131–141. (10.1007/s12042-008-9012-3)

[46] Neves SS. 2011 *Eleusine*. In Wild crop relatives: genomic and breeding resources (ed. C Kole), pp. 113–133. Berlin, Heidelberg, Germany: Springer Berlin Heidelberg. (10.1007/978-3-642-14255-0_7)

[47] Bisht MS, Mukai Y. 2001 Genomic in situ hybridization identifies genome donor of finger millet (Eleusine coracana). Theor. Appl. Genet. **102**, 825–832. (10.1007/s001220000497)

[48] Vetriventhan M, Upadhyaya HD, Dwivedi SL, Pattanashetti SK, Singh SK. 2016 Finger and foxtail millets. In Genetic and genomic resources for grain cereals improvement (eds M Singh, HD Upadhyaya), pp. 291–319. Amsterdam, The Netherlands: Elsevier. (10.1016/B978-0-12-802000-5.00007-1)

[49] Zeid M, Echenique V, Díaz M, Pessino S, Sorrells ME. 2011 *Eragrostis*. In Wild crop relatives: genomic and breeding resources (ed. C Kole), pp. 135–151. Berlin, Heidelberg, Germany: Springer Berlin Heidelberg. (10.1007/978-3-642-14255-0_8)

[50] Beldados A, Ruiz-Giralt A, Lancelotti C, Meresa Y, D’Andrea AC. 2023 Pre-aksumite plant husbandry in the horn of Africa. Veg. Hist. Archaeobot. **32**, 635–654. (10.1007/s00334-023-00949-7)

[51] Ruiz-Giralt A, Nixon-Darcus L, D’Andrea AC, Meresa Y, Biagetti S, Lancelotti C. 2023 On the verge of domestication: early use of C4 plants in the horn of Africa . Proc. Natl Acad. Sci. USA **120**, e2300166120. (10.1073/pnas.2300166120) 37364120 PMC10319037

[52] Assefa K, Tefera H, Merker A, Kefyalew T, Hundera F. 2001 Variability, heritability and genetic advance in pheno-morphic and agronomic traits of tef (Eragrostis tef (Zucc.) Trotter) germplasm from eight regions of Ethiopia. Hereditas **134**, 103–113. (10.1111/j.1601-5223.2001.00103.x) 11732845

[53] Assefa K, Merker A, Tefera H. 2003 Inter simple sequence repeat (ISSR) analysis of genetic diversity in tef [Eragrostis tef (Zucc.) Trotter]. Hereditas **139**, 174–183. (10.1111/j.1601-5223.2003.01800.x) 15061798

[54] Vadivoo AS, Joseph R, Ganesan NM. 1998 Genetic variability and diversity for protein and calcium contents in finger millet (Eleusine coracana (L.) Gaertn) in relation to grain color. Plant Foods Hum. Nutr. **52**, 353–364. (10.1023/a:1008074002390) 10426122

[55] Hiremath SC, Salimath SS. 1992 The ‘A’ genome donor of Eleusine coracana (L.) Gaertn. (Gramineae). Theor. Appl. Genet. **84**, 747–754. (10.1007/bf00224180) 24201369

[56] Hiremath SC, Salimath SS. 1991 Quantitative nuclear DNA changes in Eleusine (Gramineae). Plant Syst. Evol. **178**, 225–234. (10.1007/BF00937965)

[57] Cannarozzi G *et al*. 2014 Genome and transcriptome sequencing identifies breeding targets in the orphan crop tef (Eragrostis tef). BMC Genom. **15**, 581–581. (10.1186/1471-2164-15-581) PMC4119204 25007843

[58] Devos KM *et al*. 2023 Genome analyses reveal population structure and a purple stigma color gene candidate in finger millet. Nat. Commun **14**, 3694–3694. (10.1038/s41467-023-38915-6) 37344528 PMC10284860

[59] Olsen KM, Wendel JF. 2013 A bountiful harvest: genomic insights into crop domestication phenotypes. Annu. Rev. Plant Biol. **64**, 47–70. (10.1146/annurev-arplant-050312-120048) 23451788

[60] Pankin A, Altmüller J, Becker C, von Korff M. 2018 Targeted resequencing reveals genomic signatures of barley domestication. New Phytol. **218**, 1247–1259. (10.1111/nph.15077) 29528492 PMC5947139

[61] Kantar MB, Nashoba AR, Anderson JE, Blackman BK, Rieseberg LH. 2017 The genetics and genomics of plant domestication. BioScience **67**, 971–982. (10.1093/biosci/bix114)

[62] Liber M, Duarte I, Maia AT, Oliveira HR. 2021 The history of lentil (Lens culinaris subsp. culinaris) domestication and spread as revealed by genotyping-by-sequencing of wild and landrace accessions. Front. Plant Sci. **12**, 628439. (10.3389/fpls.2021.628439) 33841458 PMC8030269

[63] Rendón-Anaya M *et al*. 2017 Genomic history of the origin and domestication of common bean unveils its closest sister species. Genome Biol. **18**, 60. (10.1186/s13059-017-1190-6) 28356141 PMC5370463

[64] Huang X, Huang S, Han B, Li J. 2022 The integrated genomics of crop domestication and breeding. Cell **185**, 2828–2839. (10.1016/j.cell.2022.04.036) 35643084

[65] Melville J, Haines ML, Boysen K, Hodkinson L, Kilian A, Smith Date KL, Potvin DA, Parris KM. 2017 Identifying hybridization and admixture using SNPs: application of the DArTseq platform in phylogeographic research on vertebrates. R. Soc. Open Sci. **4**, 161061. (10.1098/rsos.161061) 28791133 PMC5541528

[66] Smýkal P *et al*. 2017 Genomic diversity and macroecology of the crop wild relatives of domesticated pea. Sci. Rep. **7**, 17384. (10.1038/s41598-017-17623-4) 29234080 PMC5727218

[67] Baloch FS *et al*. 2017 A whole genome DArTseq and SNP analysis for genetic diversity assessment in durum wheat from central fertile crescent. PLoS ONE **12**, e0167821. (10.1371/journal.pone.0167821) 28099442 PMC5242537

[68] Tomkowiak A, Nowak B, Sobiech A, Bocianowski J, Wolko Ł, Spychała J. 2022 The use of DArTseq technology to identify new SNP and SilicoDArT markers related to the yield-related traits components in maize. Genes **13**, 848. (10.3390/genes13050848) 35627233 PMC9142088

[69] Chaves S, Ostengo S, Bertani RP, Peña Malavera AN, Cuenya MI, Filippone MP, Castagnaro AP, Balzarini MG, Racedo J. 2022 Novel alleles linked to brown rust resistance in sugarcane. Plant Pathol. **71**, 1688–1699. (10.1111/ppa.13605)

[70] Ketema S, Tesfaye B, Keneni G, Amsalu Fenta B, Assefa E, Greliche N, Machuka E, Yao N. 2020 DArTSeq SNP-based markers revealed high genetic diversity and structured population in Ethiopian cowpea [Vigna unguiculata (L.) Walp] germplasms. PLoS ONE **15**, e0239122. (10.1371/journal.pone.0239122) 33031381 PMC7544073

[71] Adu BG, Akromah R, Amoah S, Nyadanu D, Yeboah A, Aboagye LM, Amoah RA, Owusu EG. 2021 High-density DArT-based SilicoDArT and SNP markers for genetic diversity and population structure studies in cassava (Manihot esculenta Crantz). PLoS ONE **16**, e0255290. (10.1371/journal.pone.0255290) 34314448 PMC8315537

[72] Allan V *et al*. 2020 Genome-wide DArTSeq genotyping and phenotypic based assessment of within and among accessions diversity and effective sample size in the diverse sorghum, pearl millet, and pigeonpea landraces. Front. Plant Sci. **11**, 587426. (10.3389/fpls.2020.587426) 33381130 PMC7768014

[73] Kilian A *et al*. 2012 Diversity arrays technology: a generic genome profiling technology on open platforms. In Data production and analysis in population genomics methods in molecular biology, vol. 888, pp. 67–89. Totowa, NJ: Humana Press. (10.1007/978-1-61779-870-2_5) 22665276

[74] R.Core Team. 2013 *R: a language and environment for statistical computing*. Vienna, Austria: R Foundation for Statistical Computing. See https://www.R-project.org/.

[75] Jombart T. 2008 adegenet : a R package for the multivariate analysis of genetic markers. Bioinformatics **24**, 1403–1405. (10.1093/bioinformatics/btn129) 18397895

[76] Wickham H. 2016 ggplot2: elegant graphics for data analysis. New York, NY: Springer. See https://ggplot2.tidyverse.org.

[77] Pritchard JK, Stephens M, Donnelly P. 2000 Inference of population structure using multilocus genotype data. Genetics **155**, 945–959. (10.1093/genetics/155.2.945) 10835412 PMC1461096

[78] Evanno G, Regnaut S, Goudet J. 2005 Detecting the number of clusters of individuals using the software structure: a simulation study. Mol. Ecol. **14**, 2611–2620. (10.1111/j.1365-294x.2005.02553.x) 15969739

[79] Earl DA, vonHoldt BM. 2012 Structure harvester: a website and program for visualizing structure output and implementing the Evanno method. Conserv. Genet. Resour. **4**, 359–361. (10.1007/s12686-011-9548-7)

[80] Jordahl K, Bossche JVD, Fleischmann M, Wasserman J, McBride J, Gerard J. 2020 geopandas: v0.8.1. Zenodo. (10.5281/zenodo.3946761)

[81] Goudet J. 2005 hierfstat, a package for R to compute and test hierarchical F ‐statistics. Mol. Ecol. Notes **5**, 184–186. (10.1111/j.1471-8286.2004.00828.x)

[82] Mora-García S, Yanovsky MJ. 2018 A large deletion within the clock gene LNK2 contributed to the spread of tomato cultivation from Central America to Europe. Proc. Natl Acad. Sci. USA **115**, 6888–6890. (10.1073/pnas.1808194115) 29895686 PMC6142205

[83] Ong PW *et al*. 2023 Environment as a limiting factor of the historical global spread of mungbean. eLife **12**, e85725. (10.7554/elife.85725) 37204293 PMC10299821

[84] Razzaq A, Wani SH, Saleem F, Yu M, Zhou M, Shabala S. 2021 Rewilding crops for climate resilience: economic analysis and de novo domestication strategies. J. Exp. Bot. **72**, 6123–6139. (10.1093/jxb/erab276) 34114599

[85] Combes M, Dereeper A, Severac D, Bertrand B, Lashermes P. 2013 Contribution of subgenomes to the transcriptome and their intertwined regulation in the allopolyploid Coffea arabica grown at contrasted temperatures. New Phytol. **200**, 251–260. (10.1111/nph.12371) 23790161

[86] Blischak PD, Kubatko LS, Wolfe AD. 2018 SNP genotyping and parameter estimation in polyploids using low-coverage sequencing data. Bioinformatics **34**, 407–415. (10.1093/bioinformatics/btx587) 29028881

[87] Byrne P. 2023 Teff (*Eragrostis tef*). In Understudied indigenous crops (eds K Chen, P Byrne). Fort Collins, CO: Colorado State University. See https://colostate.pressbooks.pub/understudiedindigenouscrops/chapter/importance/.

[88] Martin SH, Davey JW, Jiggins CD. 2015 Evaluating the use of ABBA-BABA statistics to locate introgressed loci. Mol. Biol. Evol. **32**, 244–257. (10.1093/molbev/msu269) 25246699 PMC4271521

[89] Ayele M, Tefera H, Assefa K, Nguyen HT. 1999 Genetic characterization of two Eragrostis species using AFLP and morphological traits. Hereditas **130**, 33–40. (10.1111/j.1601-5223.1999.00033.x) 10364827

[90] Page A, Gibson J, Meyer RS, Chapman MA. 2019 Eggplant domestication: pervasive gene flow, feralization, and transcriptomic divergence. Mol. Biol. Evol. **36**, 1359–1372. (10.1093/molbev/msz062) 31039581

[91] Wang H, Vieira FG, Crawford JE, Chu C, Nielsen R. 2017 Asian wild rice is a hybrid swarm with extensive gene flow and feralization from domesticated rice. Genome Res. **27**, 1029–1038. (10.1101/gr.204800.116) 28385712 PMC5453317

[92] Lister DL, Jones H, Oliveira HR, Petrie CA, Liu X, Cockram J, Kneale CJ, Kovaleva O, Jones MK. 2018 Barley heads east: genetic analyses reveal routes of spread through diverse Eurasian landscapes. PLoS ONE **13**, e0196652. (10.1371/journal.pone.0196652) 30020920 PMC6051582

[93] McAlvay AC, Ragsdale AP, Mabry ME, Qi X, Bird KA, Velasco P, An H, Pires JC, Emshwiller E. 2021 Brassica rapa domestication: untangling wild and feral forms and convergence of crop morphotypes. Mol. Biol. Evol. **38**, 3358–3372. (10.1093/molbev/msab108) 33930151 PMC8321528

[94] Kanatas P, Gazoulis I, Zannopoulos S, Tataridas A, Tsekoura A, Antonopoulos N. 2021 Shattercane (Sorghum bicolor (L.) Moench subsp. Drummondii) and weedy sunflower (Helianthus annuus L.)—crop wild relatives (CWRs) as weeds in agriculture. Diversity (Basel) **13**, 10.

[95] Mariac C *et al*. 2006 Genetic diversity and gene flow among pearl millet crop/weed complex: a case study. Theor. Appl. Genet. **113**, 1003–1014. (10.1007/s00122-006-0360-9) 16924479

[96] Bultosa G. 2016 Tef: overview. In Encyclopedia of food grains, 2nd edn (eds CW Wrigley, H Corke, K Seetharaman, J Faubion), pp. 209–220. Oxford, UK: Elsevier. (10.1016/B978-0-12-394437-5.00018-8)

[97] Girma D, Cannarozzi G, Weichert A, Tadele Z. 2018 Genotyping by sequencing reasserts the close relationship between tef and its putative wild Eragrostis progenitors. Diversity **10**, 17. (10.3390/d10020017)

[98] Somaratne Y, Guan DL, Abbood NN, Zhao L, Wang WQ, Xu SQ. 2019 Comparison of the complete Eragrostis pilosa chloroplast genome with its relatives in Eragrostideae (Chloridoideae; Poaceae). Plants **8**, 485. (10.3390/plants8110485) 31717580 PMC6918254

[99] Gross BL, Olsen KM. 2010 Genetic perspectives on crop domestication. Trends Plant Sci. **15**, 529–537. (10.1016/j.tplants.2010.05.008) 20541451 PMC2939243

[100] Brandt SA. 1984 New perspectives on the origins of food production in Ethiopia. In From hunters to farmers (eds JD Clark, SA Brandt), pp. 173–190. Berkeley, CA: University of California Press. (10.1525/9780520407213-021)

[101] Brandt SA. 1996 A model for the origins and evolution of *Ensete* food production. In Ensete-based sustainable agriculture in Ethiopia (eds T Abate, S Gebremariam, C Hlebsch, S Brandt), pp. 36–46. Addis Ababa, Ethiopia: Institute for Agricultural Research. See https://api.semanticscholar.org/CorpusID:134378047.

[102] Hildebrand EA. 2003 Enset, yams, and honey: ethnoarchaeological approaches to the origins of horticulture in southwest Ethiopia. [Cambridge, UK]: ProQuest Dissertations & Theses.

[103] Hildebrand E. 2007 A tale of two tuber crops: how attributes of enset and yams may have shaped prehistoric human-plant interactions in southwest ethiopia, pp. 273–298, 1st edn. London, UK: Routledge.

[104] Hildebrand E, Brandt SA, Lesur-Gebremariam J. 2010 The Holocene archaeology of southwest Ethiopia: new insights from the Kafa archaeological project. Afr. Archaeol. Rev. **27**, 255–289. (10.1007/s10437-010-9079-8)

[105] Lesur J, Vigne JD, Gutherz X. 2007 Exploitation of wild mammals in south-west Ethiopia during the Holocene (4000 BC–500 AD): the finds from Moche Borago shelter (Wolayta). Environ. Archaeol. **12**, 139–159. (10.1179/174963107x226417)

[106] Arthur JW, Curtis M, Arthur K, Coltorti M, Pieruccini P, Lesur J. 2019 The transition from hunting–gathering to food production in the gamo highlands of southern Ethiopia

[107] Arthur KW, Arthur JW, Curtis M, Lakew B, Lesur-Gebremariam J. 2009 Historical archaeology in the highlands of southern Ethiopia: preliminary findings. Nyame Akuma **72**, 3–11.

[108] Arthur KW, Arthur JW, Curtis M, Lakew B, Lesur J, Ethiopia Y. 2010 Fire on the mountain: dignity and prestige in the history and archaeology of the Borada highlands in southern Ethiopia. SAA Archaeol. Rec. **10**, 17–21.

[109] Phillipson DW. 2004 The Aksumite roots of medieval Ethiopia. Azania **39**, 77–89. (10.1080/00672700409480389)

[110] Dombrowski JC. 1970 Preliminary report on excavations in Lalibela and Natchabiet Caves, Begemeder. Ann DÉthiopie **8**, 21–29.

[111] Dombrowski JC. 1971 Excavations in Ethiopia: Lalibela and Natchabiet caves, Begemeder province, Ethiopia. [Boston, MA]: Boston University.

[112] D’Andrea AC. 2008 T’ef (Eragrostis tef) in ancient agricultural systems of highland Ethiopia. Econ. Bot. **62**, 547–566. (10.1007/s12231-008-9053-4)

[113] Varisco DM. 2023 Agricultural crops in South Arabia/Yemen in the first millennium CE. Veg. Hist. Archaeobot. (10.1007/s00334-023-00975-5)

[114] Weisskopf A, Harvey E, Kingwell-Banham E, Kajale M, Mohanty R, Fuller DQ. 2014 Archaeobotanical implications of phytolith assemblages from cultivated rice systems, wild rice stands and macro-regional patterns. J. Archaeol. Sci. **51**, 43–53. (10.1016/j.jas.2013.04.026)

[115] Gimode D, Odeny DA, de Villiers EP, Wanyonyi S, Dida MM, Mneney EE, Muchugi A, Machuka J, de Villiers SM. 2016 Identification of SNP and SSR markers in finger millet using next generation sequencing technologies. PLoS ONE **11**, e0159437. (10.1371/journal.pone.0159437) 27454301 PMC4959724

[116] Liu Q, Triplett JK, Wen J, Peterson PM. 2011 Allotetraploid origin and divergence in Eleusine (Chloridoideae, Poaceae): evidence from low-copy nuclear gene phylogenies and a plastid gene chronogram. Ann. Bot. **108**, 1287–1298. (10.1093/aob/mcr231) 21880659 PMC3197458

[117] Meresa Y, Ruiz-Giralt A, Beldados A, Lancelotti C, D’Andrea AC. 2024 Pre-Aksumite and Aksumite agricultural economy at Ona Adi, Tigrai (Ethiopia): first look at a 1000-year history. Afr. Archaeol. Rev. (10.1007/s10437-024-09574-9)

[118] Ruiz-Giralt A, Beldados A. 2024 The development of crop production in the northern horn of Africa: a review of the archaeobotanical evidence. Azania **59**, 1–30. (10.1080/0067270X.2024.2316518)

[119] Mueller NG, Goldstein ST, Odeny D, Boivin N. 2022 Variability and preservation biases in the archaeobotanical record of Eleusine coracana (finger millet): evidence from iron age Kenya. Veg. Hist. Archaeobot. **31**, 279–290. (10.1007/s00334-021-00853-y)

[120] Helm R, Crowther A, Shipton C, Tengeza A, Fuller D, Boivin N. 2012 Exploring agriculture, interaction and trade on the eastern African littoral: preliminary results from Kenya. Azania **47**, 39–63. (10.1080/0067270x.2011.647947)

[121] Robertshaw P. 1991 Gogo Falls. Azania **26**, 63–195. (10.1080/00672709109511425)

[122] Ambrose SH, Collett D, Collett D, Marshall F. 1984 Excavations at Deloraine, Rongai, 1978. Azania **19**, 79–104. (10.1080/00672708409511329)

[123] Young R, Thompson G. 1999 Missing plant foods? Where is the archaeobotanical evidence for sorghum and finger millet in East Africa? In The exploitation of plant resources in ancient Africa (ed. M van der Veen), pp. 63–72. Boston, MA: Springer US. (10.1007/978-1-4757-6730-8_6)

[124] Giblin JD, Fuller DQ. 2011 First and second millennium A.D. agriculture in Rwanda: archaeobotanical finds and radiocarbon dates from seven sites. Veg. Hist. Archaeobot. **20**, 253–265. (10.1007/s00334-011-0288-0)

[125] Kahlheber S, Höhn A, Rupp N. 2009 Archaeobotanical studies at Nok sites: an interim report. Nyame Akuma **71**, 2–17.

[126] Ruiz-Giralt A, Beldados A, Biagetti S, D’Agostini F, D’Andrea AC, Meresa Y, Lancelotti C. 2023 Sorghum and finger millet cultivation during the Aksumite period: insights from ethnoarchaeological modelling and microbotanical analysis. J. Comput. Appl. Archaeol. **6**, 96–116. (10.5334/jcaa.132)

[127] Weber SA. 1998 Out of Africa: the initial impact of millets in South Asia. Curr. Anthropol. **39**, 267–274. (10.1086/204725)

[128] Fuller DQ, Boivin N. 2009 Crops, cattle and commensals across the Indian Ocean. Études Océan Indien **42–43**, 13–46. (10.4000/oceanindien.698)

[129] Pokharia A, Srivastava C. 2013 Current status of archaeobotanical studies in Harappan civilization: an archaeological perspective. Heritage **1**, 118–137.

[130] Burgarella C *et al*. 2018 A western Sahara centre of domestication inferred from pearl millet genomes. Nat. Ecol. Evol. **2**, 1377–1380. (10.1038/s41559-018-0643-y) 30082736

[131] Wu X *et al*. 2022 Genomic footprints of sorghum domestication and breeding selection for multiple end uses. Mol. Plant **15**, 537–551. (10.1016/j.molp.2022.01.002) 34999019

[132] Fuller DQ. 2006 Agricultural origins and frontiers in South Asia: a working synthesis. J. World Prehist. **20**, 1–86. (10.1007/s10963-006-9006-8)

[133] Fuller DQ. 2011 Finding plant domestication in the Indian subcontinent. Curr. Anthropol. **52**, S347–S362. (10.1086/658900)

[134] Fuller DQ, Stevens CJ. 2018 Sorghum domestication and diversification: a current archaeobotanical perspective. In Plants and people in the african past: progress in african archaeobotany (eds AM Mercuri, AC D’Andrea, R Fornaciari, A Höhn), pp. 427–452. Cham, Switzerland: Springer International Publishing. (10.1007/978-3-319-89839-1_19)

[135] Fuller DQ, Barron A, Champion L, Dupuy C, Commelin D, Raimbault M, Denham T. 2021 Transition from wild to domesticated pearl millet (Pennisetum glaucum) revealed in ceramic temper at three middle Holocene sites in northern Mali. Afr. Archaeol. Rev. **38**, 211–230. (10.1007/s10437-021-09428-8) 34720323 PMC8550313

[136] Davey JW, Hohenlohe PA, Etter PD, Boone JQ, Catchen JM, Blaxter ML. 2011 Genome-wide genetic marker discovery and genotyping using next-generation sequencing. Nat. Rev. Genet. **12**, 499–510. (10.1038/nrg3012) 21681211

[137] Andrews KR, Good JM, Miller MR, Luikart G, Hohenlohe PA. 2016 Harnessing the power of RADseq for ecological and evolutionary genomics. Nat. Rev. Genet. **17**, 81–92. (10.1038/nrg.2015.28) 26729255 PMC4823021

[138] Blench R. 2016 Finger millet: the contribution of vernacular names towards its prehistory. Archaeol. Anthropol. Sci. **8**, 79–88. (10.1007/s12520-012-0103-6)

[139] Boivin N, Crowther A, Prendergast M, Fuller DQ. 2014 Indian ocean food globalisation and Africa. Afr. Archaeol. Rev. **31**, 547–581. (10.1007/s10437-014-9173-4)

[140] Ekstrom H, Edens C. 2003 Prehistoric agriculture in highland Yemen: new results from Dhamar. Bull. Am. Inst. Yemeni Stud **45**, 23–35.

[141] Harrower MJ, McCorriston J, D’Andrea AC. 2010 General/specific, local/global: comparing the beginnings of agriculture in the horn of Africa (Ethiopia/Eritrea) and Southwest Arabia (Yemen). Am. Antiq. **75**, 452–472. (10.7183/0002-7316.75.3.452)

[142] Dabrowski V, Tengberg M, Creissen T, Rougeulle A. 2018 Plant supplying strategies in an Islamic Omani Harbour City: archaeobotanical analysis from a workshop (B39) in Qalhāt (XIVth-XVIth c. AD). J. Islam. Archaeol. **5**, 17–38. (10.1558/jia.37690)

[143] Abraham SA. 2023 Recent developments in the archaeology of long-distance connections across the ancient Indian Ocean. Annu. Rev. Anthropol. **52**, 115–135. (10.1146/annurev-anthro-101819-110124)

[144] Ehret C. 1998 An african classical age: eastern and southern africa in world history, 1000 b.c. to a.d. 400. Charlottesville, VA: University Press of Virginia.

[145] Olatoyan J, Neumann FH, Orijemie E, Sievers C, Evans M, Mvelase S, Hatting T, Schoeman MH. 2022 Archaeobotanical evidence for the emergence of pastoralism and farming in southern Africa. Acta Palaeobot. **62**, 50–75. (10.35535/acpa-2022-0005)

[146] González-Rabanal B, Marín-Arroyo AB, Cristiani E, Zupancich A, González-Morales MR. 2022 The arrival of millets to the Atlantic coast of northern Iberia. Sci. Rep. **12**, 18589–18589. (10.1038/s41598-022-23227-4) 36329241 PMC9633756

[147] Kirleis W, Dal Corso M, Millet FD. 2022 Millet and what else? the wider context of the adoption of millet cultivation in Europe. Scales of transformation series, vol. 14, 1st edn. Leiden, The Netherlands: Sidestone Press. (10.59641/o7235ra). See https://www.sidestone.com/books/millet-and-what-else.

[148] Jones M, Hunt H, Lightfoot E, Lister D, Liu X, Motuzaite-Matuzeviciute G. 2011 Food globalization in prehistory. World Archaeol. **43**, 665–675. (10.1080/00438243.2011.624764)

[149] Liu X *et al*. 2019 From ecological opportunism to multi-cropping: mapping food globalisation in prehistory. Quat. Sci. Rev. **206**, 21–28. (10.1016/j.quascirev.2018.12.017)

[150] Pokharia AK, Sharma S, Tripathi D, Mishra N, Pal JN, Vinay R, Srivastava A. 2017 Neolithic−early historic (2500–200 BC) plant use: the archaeobotany of Ganga Plain, India. Quat. Int. **443**, 223–237. (10.1016/j.quaint.2016.09.018)

[151] Fuller DQ, Harvey EL. 2006 The archaeobotany of Indian pulses: identification, processing and evidence for cultivation. Environ. Archaeol. **11**, 219–246. (10.1179/174963106x123232)

[152] Stevens CJ, Nixon S, Murray MA, Fuller DQ. 2016 Archaeology of African plant use, vol. 61. London, UK: Routledge.

[153] Machado MJ, Pérez-González A, Benito G. 1998 Paleoenvironmental changes during the last 4000 yr in the Tigray, Northern Ethiopia. Quat. Res. **49**, 312–321. (10.1006/qres.1998.1965)

[154] Umer M, Legesse D, Gasse F, Bonnefille R, Lamb HF, Leng MJ. 2004 Late Quaternary climate changes in the Horn of Africa. (10.1007/978-1-4020-2121-3_9)

[155] Gasse F. 2000 Hydrological changes in the African tropics since the last glacial maximum. Quat. Sci. Rev. **19**, 189–211. (10.1016/s0277-3791(99)00061-x)

[156] Gebru T, Eshetu Z, Huang Y, Woldemariam T, Strong N, Umer M, DiBlasi M, Terwilliger VJ. 2009 Holocene palaeovegetation of the Tigray Plateau in northern Ethiopia from charcoal and stable organic carbon isotopic analyses of gully sediments. Palaeogeogr. Palaeoclimatol. Palaeoecol. **282**, 67–80. (10.1016/j.palaeo.2009.08.011)

[157] Butzer KW. 1981 Rise and fall of Axum, Ethiopia: a geo-archaeological interpretation. Am. Antiq. **46**, 471–495. (10.2307/280596)

[158] Brancaccio L, Calderoni G, Coltorti M, Dramis F. 1997 Phases of soil erosion during the Holocene in the highlands of western Tigray (northern Ethiopia): a preliminary report. In The environmental history and human ecology of northern Ethiopia in the late Holocene (ed KA Bard), pp. 29–44. Naples, Italy: Istituto Universitario Orientale.

[159] Berakhi O, Brancaccio L, Calderoni G, Coltorti M, Dramis F, Umer MM. 1998 The Mai Maikden sedimentary sequence: a reference point for the environmental evolution of the highlands of northern Ethiopia. Geomorphology (Amst.) **23**, 127–138.

[160] Dramis F, Umer M, Calderoni G, Haile M. 2003 Holocene climate phases from buried soils in Tigray (northern Ethiopia): comparison with lake level fluctuations in the main Ethiopian rift. Quat. Res. **60**, 274–283. (10.1016/j.yqres.2003.07.003)

[161] Marshall MH, Lamb HF, Davies SJ, Leng MJ, Kubsa Z, Umer M, Bryant C. 2009 Climatic change in northern Ethiopia during the past 17,000 years: a diatom and stable isotope record from Lake Ashenge. Palaeogeogr. Palaeoclimatol. Palaeoecol. **279**, 114–127. (10.1016/j.palaeo.2009.05.003)

[162] Meyer RS, Purugganan MD. 2013 Evolution of crop species: genetics of domestication and diversification. Nat. Rev. Genet. **14**, 840–852. (10.1038/nrg3605) 24240513

[163] Goldstein ST *et al*. 2024 Early agriculture and crop transitions at Kakapel rockshelter in the Lake Victoria region of eastern Africa. Proc. R. Soc. B **291**, 20232747. (10.1098/rspb.2023.2747) PMC11335020 38981530

[164] Borojevic K. 2011 Interpreting, dating, and reevaluating the botanical assemblage from Tell Kedesh: a case study of historical contamination. J. Archaeol. Sci. **38**, 829–842. (10.1016/j.jas.2010.11.005)

[165] Salavert A *et al*. 2020 Direct dating reveals the early history of opium poppy in western Europe. Sci. Rep. **10**, 20263. (10.1038/s41598-020-76924-3) 33219318 PMC7679390

[166] Mekonnen DZ, Gomes AI, Machado R, Oliveira HRC. 2025 Supplementary material from: The Genomics of T’ef and Finger Millet Domestication and Spread. Figshare. (10.6084/m9.figshare.c.7752361) PMC12079134 40370026

